# Supplementary material for: Two Alimentary Canal Proteins, Fo-GN and Fo-Cyp1, Act in Western Flower Thrips, Frankliniella occidentalis TSWV Infection
Source: Insects. 2023 Feb 3;14(2):154. doi: 10.3390/insects14020154 (PMC9965231; doi:10.3390/insects14020154)
Supplement: Supplementary file 1 [file insects-14-00154-s001.zip › insects-2178298-supplementary.pdf]

Table S1. Primers used in this study

Genes	Sequence (5'-3')	Uses	Annealing temperature (^0C)	Expected size (bp)
Cyclophilin	GCGATGTTGTCCCCAAGACT	RT-PCR	52.0	191
	CTGGAAGTTCTCGTCAGCGA	RT-qPCR		
T7+ Cyclophilin	TAATACGACTCACTATAGGGAG GCGATGTTGTCCCCAAGACT	RNAi	52.0	237
	TAATACGACTCACTATAGGGAG CTGGAAGTTCTCGTCAGCGA			
Glycoprotein	GTCAAGTCCGTCCCCCAGTA	RT-PCR	55.0	312
	GTCGAACTCCTGAACCTGGC	RT-qPCR		
T7+ Glycoprotein	TAATACGACTCACTATAGGGAG GTCAAGTCCGTCCCCCAGTA	RNAi	55.0	358
	TAATACGACTCACTATAGGGAG GTCGAACTCCTGAACCTGGC			
Elongation Factor 1	TCA AGG AAC TGC GTC GTG GAT	RT-qPCR	52.0	160
	ACA GGG GTG TAG CCG TTA GAG			
